# The Spatial Heterogeneity between Japanese Encephalitis Incidence Distribution and Environmental Variables in Nepal

**DOI:** 10.1371/journal.pone.0022192

**Published:** 2011-07-21

**Authors:** Daniel E. Impoinvil, Tom Solomon, W. William Schluter, Ajit Rayamajhi, Ram Padarath Bichha, Geeta Shakya, Cyril Caminade, Matthew Baylis

**Affiliations:** 1 LUCINDA Group, Department of Epidemiology and Population Health, Institute of Infection and Global Health, University of Liverpool, Leahurst Campus, Neston, Cheshire, United Kingdom; 2 Brain Infections Group, Department of Clinical Infection, Microbiology and Immunology, Institute of Infection and Global Health, University of Liverpool, Liverpool, Merseyside, United Kingdom; 3 Programme for Immunization Preventable Diseases, World Health Organization Country Office for Nepal, Kathmandu, Nepal; 4 Department of Pediatrics, National Academy of Medical Sciences, Kanti Children's Hospital, Maharajgunj, Kathmandu, Nepal; 5 Child Health Division, Department of Health Services, Ministry of Health and Population, Government of Nepal, Kathmandu, Nepal; 6 National Public Health Laboratory, Department of Health Services, Ministry of Health and Population, Government of Nepal, Kathmandu, Nepal; 7 Department of Earth's Changing Environment, School of Environmental Science, University of Liverpool, Liverpool, Merseyside, United Kingdom; University of Hong Kong, Hong Kong

## Abstract

**Background:**

To identify potential environmental drivers of Japanese Encephalitis virus (JE) transmission in Nepal, we conducted an ecological study to determine the spatial association between 2005 Nepal JE incidence, and climate, agricultural, and land-cover variables at district level.

**Methods:**

District-level data on JE cases were examined using Local Indicators of Spatial Association (LISA) analysis to identify spatial clusters from 2004 to 2008 and 2005 data was used to fit a spatial lag regression model with climate, agriculture and land-cover variables.

**Results:**

Prior to 2006, there was a single large cluster of JE cases located in the Far-West and Mid-West terai regions of Nepal. After 2005, the distribution of JE cases in Nepal shifted with clusters found in the central hill areas. JE incidence during the 2005 epidemic had a stronger association with May mean monthly temperature and April mean monthly total precipitation compared to mean annual temperature and precipitation. A parsimonious spatial lag regression model revealed, 1) a significant negative relationship between JE incidence and April precipitation, 2) a significant positive relationship between JE incidence and percentage of irrigated land 3) a non-significant negative relationship between JE incidence and percentage of grassland cover, and 4) a unimodal non-significant relationship between JE Incidence and pig-to-human ratio.

**Conclusion:**

JE cases clustered in the terai prior to 2006 where it seemed to shift to the Kathmandu region in subsequent years. The spatial pattern of JE cases during the 2005 epidemic in Nepal was significantly associated with low precipitation and the percentage of irrigated land. Despite the availability of an effective vaccine, it is still important to understand environmental drivers of JEV transmission since the enzootic cycle of JEV transmission is not likely to be totally interrupted. Understanding the spatial dynamics of JE risk factors may be useful in providing important information to the Nepal immunization program.

## Introduction

Japanese Encephalitis (JE) is a viral zoonotic mosquito-borne disease which causes 30,000 to 50,000 human cases per year, primarily in Asia, making it the most common cause of viral encephalitis [1]. Infection results in case fatalities of 0.3% to 60% [2], [3]. JE is caused by the JE virus (JEV), a member of the Flaviviridae family, which also includes dengue virus, West Nile virus, and Yellow fever virus [4]. The virus is transmitted through a wide range of vectors, but the primary vector of JEV across Asia is the mosquito *Culex tritaeniorhynchus*. JEV infects a wide range of vertebrates; however its primary reservoir host is water birds such as egrets and herons [5]. Rice-fields are the preferred development sites for *C. tritaeniorhynchus* as well as the main foraging site for water birds; thus rice-fields provide an important transmission site for infectious and susceptible birds and mosquitoes to meet. Pigs are considered a secondary reservoir host because they produce high viraemias when infected, and readily infect pig-biting mosquitoes. Humans are considered tangential hosts because low viremia is produced when they are infected, which means they cannot infect mosquitoes when bitten; hence, the virus transmission cycle reaches an end when humans are infected. When mosquito populations become adequately high, JEV transmission spills over from the mosquito-bird-pig cycle into human populations. Two major risk factors of JEV transmission to humans are close proximity to rice fields and pigsties.

Major environmental drivers capable of amplifying JEV transmission are temperature and precipitation, which act primarily through their influence on mosquito vector life history such as development time of immature mosquito stages (i.e. larvae and pupae) and population abundance [6], [7], [8], [9], [10] and the extrinsic incubation period of the virus in the mosquito once it is acquired by bite from an infected vertebrate host [11]. However, with the concern of climate change threatening increased transmission of several arboviruses [12], advancing the knowledge base of JE-climate interactions is needed. Early studies of JE epidemiology demonstrated that JEV transmission was associated with high temperature and low rainfall [13]. Other studies in China [14] and Taiwan [15] have found significant temporal associations between climate and JEV transmission with different time lags. Despite these studies, a dearth of spatial studies exists that examine the associations between JEV transmission and climate variables. Other potential drivers of JEV transmission include land-use and land-cover [16], [17], [18].

While the influence of environmental drivers of JEV transmission has been described in other countries across Asia, few have investigated these drivers in Nepal, despite the heavy disease burden of JEV infection among human populations. JE was first recognized in Nepal in the terai region in 1978. The terai region is comprised of a belt of marshy grasslands, savannas, and forests at the base of the Himalayan Mountains. The altitude ranges from approximately 55 to 500 m. The transmission season in Nepal starts from June to October with a peak in August [19]. High case-fatality (15%–35%) due to JEV transmission has been reported in the terai since its discovery, making it the most common cause of encephalitis and a growing public health concern in the Nepal terai region [5]. Additionally, since 1997 there have been reports of JEV transmission expanding from the terai region to the hill region (altitude of 1,300 m) of Nepal with a significant number of cases appearing in the Kathmandu Valley [5], [20], [21]. It is not clear what is driving this expansion. Recent studies in animals in Nepal have found significant exposure to JEV in pigs, horses and ducks [22], [23].

From 2004 to present, Nepal has been actively detecting JE cases through their national vaccine preventable diseases surveillance network for acute encephalitis syndrome (AES) conducted by the government of Nepal, with technical and financial support from the World Health Organization (WHO) [20]. Through the national surveillance program it has been confirmed that significant transmission of JEV is indeed occurring in the Kathmandu valley [21] and other locations in the hill and mountain districts [20] of Nepal and not only the terai region of Nepal. Given these findings, it has been suggested that the national JE prevention and control program should not only focus on the terai region, but should also give attention to other regions of Nepal [20]. In addition, under the Nepal Ministry of Health and Population, mass JE vaccination campaigns have been conducted annually in a phase-wise manner targeting 1 to 8 endemic districts each year beginning in 2006 with reported coverage >80% of the targeted population. Approximately half of the 23 districts have targeted only children 1 to 15 years of age, whereas the other districts have targeted all persons greater than 12 months of age. With the current surveillance program in place, it is now possible to examine, country-wide, the spatial patterns of JEV transmission in Nepal.

The expansion of JEV transmission into the hill and mountain regions raises questions on the ecology of transmission among the various regions of Nepal and the spatial patterns therein. In this study, we look at the changing trend of clustering of JE cases in the different districts of Nepal. In addition, in this study, we focus our environmental analysis on the 2005 JEV epidemic. This is due to the large number of cases that occurred in that year (669 laboratory-confirmed cases). Furthermore, we wanted to determine the impact of climate and land-use land-cover variables on JE cases without the influence of mass immunization. We conduct the first ecological spatial analysis in Nepal to determine the spatial association between Nepal JE incidence and two climate variables (mean monthly temperature and mean total precipitation) as well as two agricultural variables (percentage irrigated land, pig-to-human ratio) and three land cover variables (deciduous tree cover, grassland cover and cropland cover) at the district level using national laboratory-confirmed data. We tested the following hypotheses: 1) the environmental drivers of JEV transmission were the same before and after the 2005 epidemic, 2) lagged monthly temperature and precipitation variables predict JE incidence better than annual climate variables and 3) in Nepal, JEV transmission is related to agricultural, land-cover and land-use patterns (i.e. pig-to-human ratio, percentage irrigated land and deciduous tree cover, grassland cover and cropland cover) that are associated with transmission cycles involving the life history of the mosquito vector and availability of the reservoir host.

## Methods

### Japanese Encephalitis case detection

JE case detection in Nepal has been described previously [24]. Briefly, the Nepal model for JE case surveillance is based on experience implementing clinical surveillance of encephalitis. Because the symptoms of JE are similar to many other encephalitic diseases, patients are initially investigated as Acute Encephalitis Syndrome (AES) patients with the etiology of the encephalitis determined later. AES cases are defined as any patient presenting with acute onset of fever and a deterioration in mental status (e.g., confusion, disorientation, coma, or inability to talk) and/or new onset of seizures excluding simple febrile seizures. Using a structured reporting form, information on age, gender, district of residence, and whether the patient had been immunized against JEV are recorded. Clinical data including date of disease onset and symptoms (e.g., fever, neck rigidity, convulsions) as well as outcome at discharge (i.e., cured, referred, death, unknown) are recorded. Five mL of serum or 2 mL of cerebrospinal fluid (CSF) are obtained from each patient. The serum or CSF samples are labeled with the patient identification number and stored at 2°C to 8°C until transported in cold boxes with ice packs to the National Public Health Laboratory in Kathmandu. Laboratory confirmation is made from a single serum or CSF sample by detection of anti-JE immunoglobulin M (IgM) antibody titers using an IgM antibody capture enzyme- linked immunoassay.

### Geographical and Meteorological data

District-level geographical information systems (GIS) shapefiles of Nepal were acquired from the International Centre for Integrated Mountain Development (geoportal.icimod.org/Downloads/) (See Figure S1. for a general map of Nepal districts). Nepal temperature and precipitation data were acquired from WorldClim Global dataset www.worldclim.org/). WorldClim is a set of global climate layers (climate grids) with a spatial resolution of a square kilometer. The WorldClim dataset, which spans from 1950 to 2000, is meant to serve as a high resolution source of climate data, which is interpolated from various climate sources [25]. The WorldClim station network over India – Nepal is relatively dense to have confidence in the final high resolution gridded product. Long-term climate normals were acquired for annual mean temperature and precipitation, as well as mean temperature and precipitation for the individual months of April and May, as well as the three month period from June to August. The WorldClim dataset is gridded and zonal estimation was used to summarize the values of the gridded climate datasets within Nepal district shapefiles using the Spatial Analyst module in ArcView GIS 9.3 ® (ESRI, Redlands, CA). In subsequent analysis we made the assumption that using the WorldClim dataset that spans from 1950 to 2000 would be able to predict JE incidence in the 2005 outbreak in Nepal.

### Agricultural and Land-use Land-cover data

Nepal agricultural data (i.e. irrigated land area and pig number) was acquired from the Nepal Ministry of Agriculture and Cooperatives (www.moac.gov.np/home/statistics.php). All data were reported as “per district” and is based on 2008/2009 estimate from surveys. We assumed no major changes in pig number and irrigated land area in each of the districts from 2004 to 2008. We calculated the percent of irrigated land for each district by taking the total irrigated land area and dividing it by the total district areas size and multiplying by one hundred. We also calculated the ratio of pig-to-human population per district to account for differences in exposure in areas with high and low human and pig populations. Land-cover data was downloaded from Boston University's MODIS Land Cover and Land Cover Dynamics project (www-modis.bu.edu/landcover/). We used the dataset consisting of the International Geosphere Biosphere Programme (IGBP) classification scheme which is comprised of 17 land cover classes. After conducting a Pearson's coefficient correlation analysis with all land cover variables and 2005 district level JE incidence values, we selected the percentage of grassland cover (r = −0.690, P<0.001) and the cropland cover (r = 0.609, P<0.001) for further analysis because they had the highest negative and positive correlation with JE incidence respectively. We also included percentage of deciduous tree cover (r = 0.442, P<0.001), since other studies suggested this may be an important factor in mosquito niche selection [26].

### Cluster analysis

Univariate Local Indicators of Spatial Association (LISA) were used to describe the spatial pattern of JE incidence clusters at the district level for the years of 2004 to 2008. LISA maps are particularly useful to assess the hypothesis of spatial randomness and to identify local hot and cold spots of a phenomenon [27], [28]. The univariate LISA gives an indication of the degree of linear association (positive or negative) between the values for one value at a given location and the average of neighboring values in the surrounding location. Univariate LISA suggest two classes of positive spatial correlation, or spatial clusters (High-High and Low-Low), and two classes of negative spatial correlation, or spatial outliers (High-Low and Low-High). The LISA cluster maps indicate only the center of the cluster. The actual extent of the cluster includes the center and the surrounding neighbors as defined by a weights matrix. In GeoDa alpha release 0.9.8.14 (geodacenter.asu.edu/software/downloads), a randomization approach is used to generate a spatially random reference distribution to assess statistical significance. In order to do spatial analysis such as LISA analysis or other spatial analyses, a contiguity-based spatial weight matrix must be created to identify spatial relationships between geographic features, in our case Nepal district polygons. In this analysis, we used a first order Rook Contiguity which uses a common boundaries of polygons to define Nepal district polygon neighbors [27]. For 2005 and 2008 data, we also calculated the relative risk of JE incidence in high JE-incident clustered areas versus non-clustered areas using SatScan™. SatScan™ uses a variable-sized window to test for possible clusters noting the number of observed and expected observations inside the window at each location [29]. To do this purely spatial analysis, we used a circular spatial window and scanned for clusters with high rates using the discrete Poisson model.

### Statistical model

In our analysis, we used a strategy similar to Wimberly et al., 2008 [30]. We used a three-step process to develop a model that identifies ecologically-important variables that are likely drivers of JEV transmission in Nepal using 2005 data only. The rationale of using this three-step process was first, to characterize how temperature and precipitation are associated with the distribution of JE cases, since these variables can have differential impacts on mosquito populations and subsequent virus transmission. For example, laboratory studies have found that there is an ‘optimum range’ for virus transmission in mosquitoes [11]. This can be modeled with quadratic polynomial terms; second, to expand the climate model to include agricultural and land-cover variables that have previously been identified as important risk factors of JEV transmission; third, to develop a parsimonious model that emphasizes the variables which are the most important for describing JEV distribution. In all models, a spatial lag regression model was done to account for spatial autocorrelation. The spatially lagged regression models determine whether the dependent variable covaries with its geographic neighbors [31]. The importance of accounting for spatial autocorrelation is to avoid spurious associations between dependent and independent variables when they share the same spatial clustering or are uniform in space [31].

Our first step was to compare competing models based on the climate variables mean temperature and mean precipitation. The models allowed for a) an interaction term between temperature and precipitation to determine if there were different responses between JE incidence and temperature for different levels of precipitation, b) a curvelinear or unimodal relationship between temperature and precipitation and JE incidence using a second-order trend surface of mean monthly temperature and mean monthly precipitation to determine if these climate parameters would provide a better fit to JE incidence and, c) a spatial lag term to account for spatial autocorrelation among the Nepal districts (i.e. account for JE clustering). Six models were compared based on the following:

1. Annual mean temperature and precipitation from 1950 to 2000
2. April mean monthly temperature and precipitation (2-month lag from transmission season)
3. May mean monthly temperature and precipitation (1-month lag from transmission season)
4. April temperature (2-month lag) and May precipitation (1-month lag)
5. April precipitation (2-month lag) and May temperature (1-month lag)
6. June-July-August mean temperature and precipitation (these 3 months represent the climate at peak JEV transmission)

The model used is below:
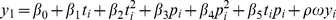
where *i* represents each Nepal district, *y_i_* is the JE incidence, *t_i_* is the temperature variable, *p_i_* is the precipitation variable, *β* is the parameter estimate and *ρω_i_y_i_* is the spatial lag term [31].

The months of April and May were chosen to encompass 2-month and 1-month climate lags respectively from the JEV transmission season which starts in June. The months of June to August were chosen since this is the time at which JEV transmission increases and peaks.

Using GeoDa, a spatial lag model approach was used to model JE incidence as a function of the competing sets of climate variables. Within each district, the incidence of JE was calculated by taking the number of JE cases in 2005 and dividing it by the 2005 population estimate for each district. District population estimateswere calculated by regressing a line through the projected Nepal population point estimates for 2001, 2006, 2011, 2016, and 2021; (www.cbs.gov.np/Gis_Maps/Population%20Projection_Trends/Population%20Projection%202001%20-%202021.gif) from the regression line the population estimates were derived. Since these projections use 2001 Nepal census data as a baseline and extrapolate their projections based on population growth estimates, we found this approach to be satisfactory in providing estimates of the district population size. Due to the sizable variation of area and population density across these administrative units, the crude 2005 incidence rates for each district polygon was first estimated by using spatial empirical Bayes smoothing method available in GeoDa. This smoothing procedure was suggested to adjust for the potential bias resulting from estimating from a small population at risk [32]. The first order Rook contiguity-based spatial weight matrix method, mentioned in the cluster analysis section, was used for the both the spatial empirical Bayes smoothing and the spatial lag model. In order to avoid the violation of the constant variance assumption in linear regression, we used a negative-reciprocal transformation on the Spatial Empirical Bayes smoothed JE incidence. This drastically reduced heteroscedasticity (i.e. irregular variance) more than a log transformation. The dependent variable (i.e. smoothed JE incidence) was transformed using a negative reciprocal (−1/(y_i_+1)) transformation. To further increase interpretability we added 1 to the newly transformed variable to make the values positive. To improve model convergence and interpretation, and reduce problems with collinearity (i.e. high correlation between independent variables) between second order and interaction terms, independent variables were centered by subtracting their mean [33]. The R^2^, adjusted R^2^ and Akaike's information criterion (AIC) were used as metrics to compare the candidate models. The model with the highest R^2^ and lowest AIC was selected as having the best fit. To assist in visualizing the relationship between JE incidence and environmental variables, we made contour plots based on the spatial lag model in Microsoft ® Excel 2007 (Microsoft, Redmond, WA).

In the second step of our analysis, a full model using the 2005 data was created by adding the agricultural, land-use and land-cover variables (i.e. pig-to-human ratio, percentage irrigated land, percentage deciduous tree cover, percentage grassland cover and percentage cropland cover) to the best fitting temperature/precipitation model. Early exploratory analysis suggested that the addition of squared term of pig-to-human population ratio may improve subsequent linear regression models. To avoid problems with collinearity, we centered pig-to-human population ratio subtracting the mean from the variable. The new centered variable and the quadratic term of the new centered variable was entered into the model. We also found that taking the natural log of percentage irrigated land, deciduous tree cover, grassland cover and cropland cover also stabilized the variance between these variables and JE incidence.

In the third and final step of our analysis, a parsimonious model was created in IBM® SPSS® Statistics 17 using a backward selection method. The subsequent model variables found to be important in IBM® SPSS® Statistics 17 (IBM SPSS, Chicago, IL) were used to create the spatial lag model in GeoDa. This was done because at present there is no backward selection method in GeoDa and it would have been computationally tedious to execute this method using partial F tests with GeoDa.

To test whether environmental driver trends of 2005 still remain constant, despite the changes in JE incidence cluster patterns due to mass vaccination programs, we used the variables in the 2005 parsimonious model to create subsequent models using 2004, 2006, 2007 and 2008 JE incidence data. We evaluated the respective models by comparing the R^2^ values and parameter estimates of 2005 with the other years.

## Results

### Seasonal transmission of JE

**Figure 1:**
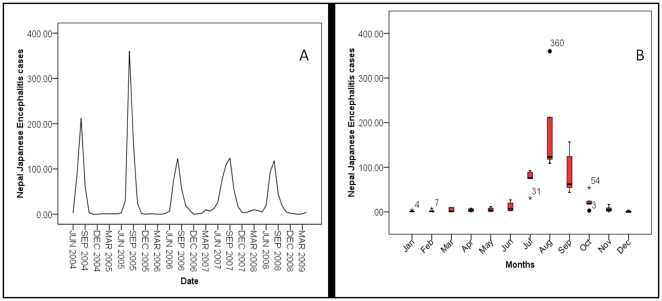
Time Series graph (A) of laboratory confirmed Japanese Encephalitis (JE) cases and a box plot (B) of seasonal transmission of confirmed JE cases from June 2004 to April of 2009. The dark line in the middle of the boxes is the median value; the bottom and top of the boxes indicates the 25th and 75th percentile respectively; whiskers represents 1.5 times the height of the box; and dots with numbers represent value of outlier cases and asterisks with number represent extreme values outlier cases (i.e. more than three times the height of the boxes).

Figure 1. shows a time series of laboratory-confirmed JE cases from June 2004 to April 2009 and the annual seasonal transmission pattern of laboratory-confirmed JE cases. Figure 1A. shows that in 2006 there was a drop in the number of JE cases. However, a substantial number of JE cases still occurred (over 100 laboratory confirmed cases across all years). Figure 1B. confirms the general trend of JEV transmission is an initial increase of transmission from June followed by a peak in August and a decrease in transmission thereafter.

### Cluster analysis

**Figure 2:**
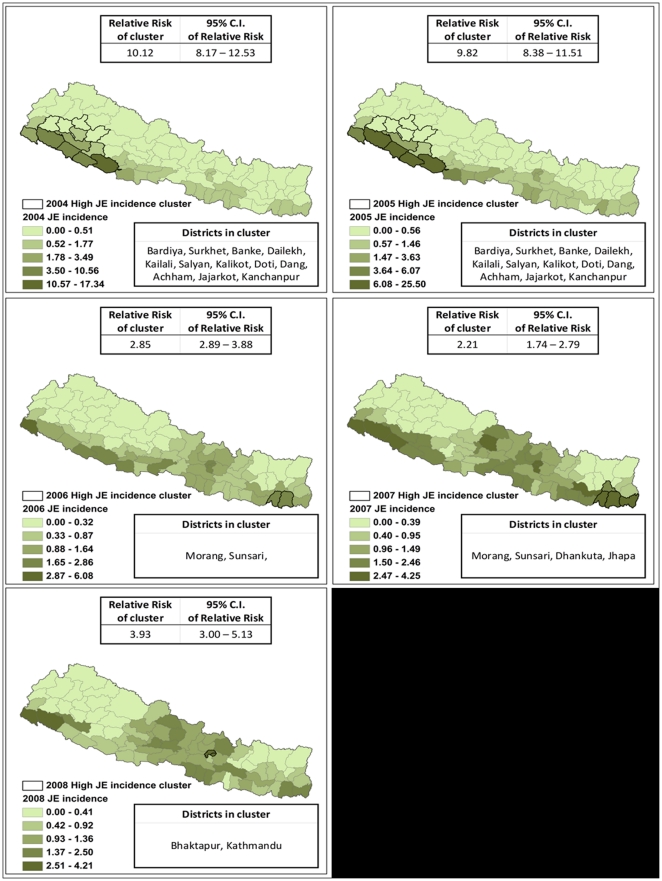
Japanese Encephalitis incidence (2004–2008) in Nepal. Spatial Empirical Bayes smoothed JE incidence expressed as the number of cases per 100,000. Maps of 2004 to 2008 show the dominate district JE cluster, the relative risk of inside the cluster to outside the cluster and 95% confidence using SatScan.

**Figure 3:**
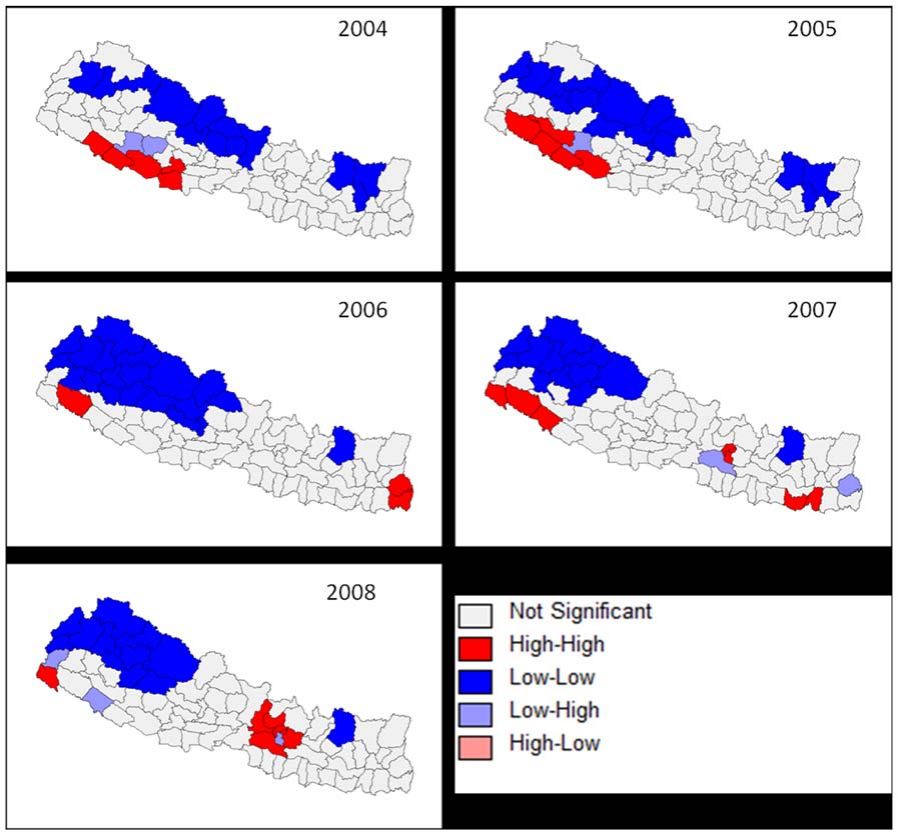
Univariate Local Indicators of Spatial Association (LISA) cluster maps from 2004 to 2008 (A–E) for JE incidence using GeoDa. The cluster map only shows the center of the cluster in color. High-High represents clustering of high JE incidence values, Low-Low represents clustering of low JE incidence values, Low-High represent low values of JE incidence clustered around high values of JE incidence, and High-Low represents high values of JE incidence clustered around low values of JE incidence.

Figure 2. shows the distribution of JE incidence and the most likely clusters in Nepal from 2004 to 2008 using SatScan. The general trend shows JEV transmission concentrated in the Mid-West and Far-West terai regions with high values of JE incidence from 2004 to 2005 but gradually decreasing in the terai region. LISA analysis of JE epidemics that occurred from 2004 to 2008 identified different foci of JE transmission (Figure 3). The shift can be seen when looking at the JE incidence distribution of the 2005 outbreak year and the later year of 2008 (Figure 3), where high values in 2008 cluster around the Kathmandu valley. Using SatScan, we found that in 2005 the relative risk for populations inside the JE cluster (which included the following districts: Achham, Banke, Bardiya, Dailekh, Dang, Doti, Jajarkot, Kailali, Kalikot, Kanchanpur, Salyan, and Surkhet; see Figure 2.) compared to those outside the cluster was 9.82 (95% Confidence interval: 8.83–11.51). While in 2008, we found that the relative risk for populations inside the JE cluster (which included the following districts: Bhaktapur and Kathmandu) compared to those outside the cluster was 3.93 (95% Confidence Interval: 3.00–5.13 see Figure 2.). This suggests higher risk of JE in these clustered areas versus outside of these clusters.

### Statistical modeling

**Figure 4:**
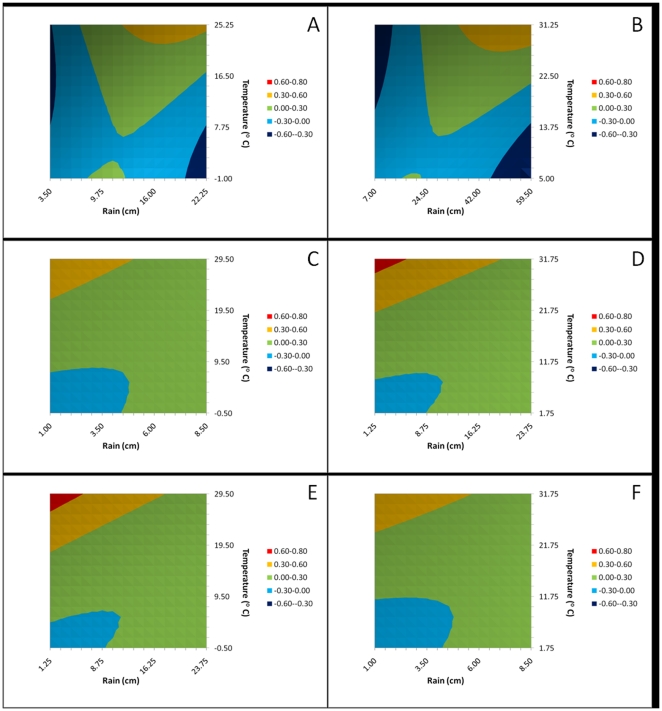
JE incidence as a function of climate variables. The response is the negative reciprocal of JE incidence. A) Annual climate model, B) June–July–August model, C) April climate model, D) May climate model, E) May temperature/April precipitation climate model and, F) April temperature/May precipitation climate model.

The model based on May temperature and April precipitation had the best fit as indicated by the high R^2^ value and low AIC (Table 1.). Temperature and the spatial lag term were the only variables found to be highly significant with a positive association with JE incidence. Quadratic and the interaction terms did not have high statistical significance. The other models had weaker fits, though the model with April temperature and precipitation was comparable in R^2^ value and AIC. Though precipitation was not statistically significant, the 2-months and 1-month lag (April and May) models did show a negative relationship with JE incidence, with high number of JE cases occurring when there is low precipitation in the preceding months (Figure 4C–F.). In the Annual and June–July–August models, precipitation had a positive, albeit non-statistically significant, association with JE incidence (Table 1. and Figure 4A and B.).

**Table 1:** Trend surface models of 2005 district-level JE incidence as a function of temperature and precipitation.

	Model β coefficients†					
Variable	Annual	Apr	May	Jun–Jul–Aug	Apr/May	May/Apr
**Intercept**	0.0528*	0.0489	0.0607*	0.0651**	0.0590*	0.0497
***t***	0.0128***	0.0101***	0.0131***	0.0132***	0.0128***	0.0109***
***t^2^***	0.0006	0.0005*	0.0008**	0.0002	0.0007**	0.0006*
***p***	0.0085	−0.0095	−0.0049	0.0036	−0.0062	−0.0066
***p^2^***	−0.0036*	0.0028	0.0004	−0.0004**	0.0005	0.0020
***pt***	0.0021	−0.0029*	−0.0012*	0.0012*	−0.0012*	−0.0029*
***ρ***	0.7942***	0.7531***	0.7307***	0.7737***	0.7366***	0.7464***
**R^2^**	0.8072	0.8207	0.8170	0.8113	0.8168	0.8213
**Adjusted R^2^**	0.7932	0.8077	0.8037	0.7976	0.8035	0.8084
**AIC**	−83.3025	−90.7539	−90.2047	−85.9352	−89.8923	−91.3228

Apr/May = April temperature/May precipitation, May/Apr = May temperature/April precipitation, ***t*** = mean temperature, ***p*** = mean precipitation, ***Rho*** (***ρ***) = spatial lag parameter.

*statistically significant at α-value 0.1,

**statistically significant at α-value 0.05,

***statistically significant at α-value 0.01.

† All regression β coefficients represent a non-linear increase (or decrease when the coefficient value is negative) in JE incidence when there is a 1-unit increase in each respective predictor variable.

Compared to the crude climate model (i.e. May temperature/April precipitation alone), higher R^2^ and higher AIC were obtained after incorporating the variables pig-to-human ratio and its quadratic term, the natural log-percentage of irrigated land and deciduous tree cover, grassland cover and cropland cover into the model (see Figure S2.). for the distribution of environmental variable values in Nepal used in full and parsimonious models). However, the only variable which was statistically significant in the full model was the interaction term and the spatial lag term (Table 2.). There was high collinearity in the full model, specifically with temperature (Variance Inflation Factor >16; see Table S1 for the Pearson's correlation coefficient matrix of the variables used in the full model for 2005 JE incidence). Therefore, we developed a parsimonious model using backward selection method in which precipitation, the percentage of irrigated land, the percentage of grassland cover and the pig-to-human ratio remained in the model, but not temperature, its quadratic term or the quadratic term of precipitation (Table 3.). The parsimonious model was found to be comparable to the full model in the R^2^ value, but the parsimonious model had a lower AIC than the full model indicating a better explanatory model. Precipitation was negatively associated with JE incidence, while percentage of irrigated land was positively associated with JE incidence. Neither the percentage of grassland cover, pig-to-human ratio nor its quadratic term was significantly associated with JE incidence.

**Table 2 pone-0022192-t002:** Full model of 2005 district-level JE incidence as a function of climate, agriculture and land-use.

Variable	β Coefficient†	S.E.	95% Confidence Interval (β)		P-value
**Intercept**	0.124	0.068	−0.009	0.258	0.068
**May – ** ***t***	0.004	0.007	−0.008	0.017	0.501
**May – ** ***t^2^***	0.001	0.001	0.000	0.001	0.137
**April – ** ***p***	−0.008	0.015	−0.037	0.021	0.581
**April – ** ***p^2^***	0.001	0.004	−0.007	0.010	0.738
***pt***	−0.004	0.002	−0.007	0.000	0.031
***Irrigated***	0.013	0.017	−0.020	0.047	0.431
***Pig-human***	0.586	0.529	−0.450	1.622	0.267
***Pig-human^2^***	−7.180	3.962	−14.945	0.585	0.070
***Deciduous***	0.000	0.036	−0.070	0.070	0.999
***Grassland***	−0.031	0.033	−0.095	0.033	0.347
***Cropland***	0.000	0.025	−0.049	0.049	0.996
***ρ***	0.682	0.088	0.509	0.855	<0.001
**R^2^**	0.830				
**Adjusted R^2^**	0.800				
**AIC**	−85.762				

***t*** = mean temperature, ***p*** = mean precipitation, ***Irrigated*** = natural log of percentage of irrigated land per district, ***Pig-human*** = pig-to-human ratio, ***Deciduous*** = natural log of percentage deciduous tree cover per district, ***Grassland*** *= *natural log of percentage grassland cover per district, ***Cropland*** = natural log of percentage cropland cover per district, ***Rho (ρ)*** = spatial lag parameter.

**S.E.** = Standard error of â coefficient.

† All regression β coefficients represent a non-linear increase (or decrease when the coefficient value is negative) in JE incidence when there is a 1-unit increase in each respective predictor variable.

**Table 3 pone-0022192-t003:** Parsimonious model of 2005 district-level JE incidence as a function of climate, agriculture and land-use.

Variable	β Coefficient†	S.E.	95% Confidence Interval (β)		Probability
**Intercept**	0.292	0.084	0.128	0.457	<0.001
**April-** ***p***	−0.032	0.009	−0.051	−0.014	<0.001
***Pig-human***	0.637	0.516	−0.374	1.648	0.217
***Pig-human^2^***	−6.053	3.856	−13.610	1.505	0.117
***Irrigated***	0.033	0.012	0.010	0.056	0.005
***Grassland***	−0.033	0.022	−0.077	0.010	0.134
***ρ***	0.689	0.086	0.520	0.859	<0.001
**R^2^**	0.8122				
**Adjusted R^2^**	0.7986				
**AIC**	−89.933				

***p*** = mean precipitation, ***Irrigated*** = natural log of percentage of irrigated land per district, ***Pig-human*** = pig-to-human ratio, ***Grassland*** = natural log of percentage grassland cover per district, **Rho (** ***ρ*** **)** = spatial lag parameter.

**S.E. = **Standard error of â coefficient.

† All regression β coefficients represent a non-linear increase (or decrease when the coefficient value is negative) in JE incidence when there is a 1-unit increase in each respective predictor variable.

When we compared the observed JE incidence with the predicted JE incidence we found that the model heavily underestimated the JE incidence in Bardiya, Banke, Dang and Kailali (observed: 25.50, 21.69, 17.25 and 15.17 vs. predicted: 2.80, 2.92, 2.25 and 1.99 respectively). However a similar pattern in JE incidence distribution was still seen between the observed and predicted maps (see Figure S3. for the predicted smoothed JE incidence in each of the districts and the prediction error using the parsimonious model).

When we applied our parsimonious model to the other years, we found that 2004, 2006 and 2007, R^2^ values, AIC, parameter coefficients were similar. Significance level among the environmental variables showed variability from year to years (Table 4.). For example, April precipitation was statistically significant during 2004 and 2005, percentage irrigated land was significant in 2005 and 2007 and percentage grassland cover was significant in 2004 and 2006. In 2007, a statistically significant unimodal relationship between JE incidence and pig-to-human ratio emerged. In 2008, April precipitation took on a positive trend, albeit not significant. The only variable consistently significant throughout the years was the spatial lag term.

**Table 4:** Comparison of parsimonious 2004 to 2008 models of district-level JE incidence as a function of climate, agriculture and land-use.

	Model β coefficient†				
Variable	2004	2005	2006	2007	2008
**Intercept**	0.2943**	0.2924**	0.2026**	0.1533*	0.1037
**April – ** ***p***	−0.0361**	−0.0323**	−0.0132	−0.0070	0.0012
***Irrigated***	0.0223*	0.0331**	0.0302**	0.0281**	0.0181
***Grassland***	−0.0497*	−0.0332	−0.0341**	−0.0221	−0.0229
***Pig-human***	0.3403	0.6372	0.5790	0.8329	0.0781
***Pig-human^2^***	−2.1978	−6.0526	−5.6163	−8.5296*	−2.0550
***ρ***	0.7330**	0.6892**	0.6952**	0.7801**	0.7725**
**R^2^**	0.8476	0.8122	0.8415	0.8305	0.7535
**Adjusted R^2^**	0.8366	0.7986	0.8300	0.8182	0.7356
**AIC**	−105.0710	−89.9330	−123.1960	−103.8150	−97.3233

*p* = mean precipitation, *Irrigated* = natural log of percentage of irrigated land per district, *Pig-human* = pig-to-human ratio, *Grassland* = natural log of percentage grassland cover per district, *ρ* = spatial lag parameter.

*statistically significant at α-value 0.05,

**statistically significant at α-value 0.01.

† All regression β coefficients represent a non-linear increase (or decrease when the coefficient value is negative) in JE incidence when there is a 1-unit increase in each respective predictor variable.

All regression β coefficients in Table 1–4 represent a non-linear increase (or decrease when the coefficient value is negative) in JE incidence when there is a 1-unit increase in each respective predictor variable.

## Discussion

In this paper we conducted one of the first spatial analyses of JEV transmission in Nepal. Using laboratory confirmed JE cases from the national AES surveillance program in Nepal, we identified spatial clusters of JE incidence in 2004 to 2008 and developed a spatial statistical model with climate and land-cover variables. We identified key variables that describe the spatial distribution of JE cases in Nepal.

JE clustered mostly in the Far-West and Mid-West terai Regions of Nepal in 2005. In 2008, a large JE cluster was found to exist in the central hill area, primarily the Kathmandu, Lalitpur, Bhaktapur districts (i.e. Kathmandu Valley). It has been suggested that the shifts in JEV transmission distribution may be due to 1) the mass immunization campaigns conducted in Nepal, which have been introduced since 2006 and are being conducted in a phase-wise manner starting with highest priority districts assigned by Nepal MOH or 2) conferred widespread immunity resulting in fewer susceptible people in the terai because of the large JE epidemic in 2005 [21].

However, the discovery that higher elevation locations in the hill areas, in particular the Kathmandu Valley, was found to have moderate levels of JEV transmission is a considerable finding, given that this region was not previously thought to have a significant JE problem [21]. Under conventional wisdom, the altitude (1,500 m) and subsequent lower temperature of the valley would preclude the possibility of transmission due to low likelihood the mosquito vectors could survive in this location and the temperature threshold for transmission of JEV in the mosquito could be achieved. However, studies have found potential mosquito vectors in the hill and mountain areas of Nepal [34], [35], [36]. Also, JEV transmission has been shown to still occur at temperatures as low as 20°C [11], which is a temperature that can be experienced in the Kathmandu Valley. Though JEV transmission in the Kathmandu Valley has been reported previously [37], detection was limited to only one hospital over a limited timeframe [21]. Further investigation in this area is required to understand the dynamics of transmission in this area to rule any effects of other confounding circumstances such as human movement of infected individuals into the region.

With regards to climate variables, we used the WorldClim dataset which represent climate normals over a 50-year period (1950–2000) rather than weather data at the time of the 2005 outbreak. Wimberly et al found that climate normals provided a better model fit to West Nile incidence than annual weather and weather anomalies [28], suggesting that long-term trends in weather pattern, better predicts subsequent pathogen transmission. For that reason we make the assumption that the predictors of JE incidence used in our analysis are fairly robust and climate shapes the distribution of JE incidence in Nepal. In our study, lagged monthly climate variables better predicted JE incidence rather than annual or concurrent (June–July–Aug) monthly climate variables. Districts with high temperature in the preceding months of the JEV transmission season had higher JE incidence. Similar patterns have been reported from China, Taiwan and Japan [13], [14], [15]. The rationale was that high temperature decreased development time for larval and pupal mosquito stages. However, in our study, districts with low precipitation in the preceding months of the JEV transmission season also had higher JE incidence. It has been suggested by Mogi 1983 [13] that low precipitation prevented the loss of mosquito immature stages from high water currents. Similarly, it has been suggested in the US that drought periods induce large outbreaks of West Nile virus transmission [38], [39], [40], [41]. It is thought that drought increases the association between mosquito vectors and birds by reducing the number of water sites available, hence concentrating birds and mosquitoes in one area. Alternatively, during these drought periods, predators of mosquitoes are significantly reduced; hence natural regulation of mosquito populations is significantly impeded. While this is true for WNV it is not clear if the same exists for JEV vectors. In contrast, a study in India found that rice-fields, primary sites for JEV transmission, are relatively stable ecosystems and sudden increases in mosquito populations as a function of climate are uncommon [42]. In our study, we lacked entomological data to complement the JE incidence data and given the somewhat contradictory results from US-Japan and India studies, we are unable to conclude with confidence that the associations with climate identified in Nepal occur via effects on mosquito populations. Still, the results of this study give insights into the relationship between JEV transmission and climate in Nepal.

Percentage irrigated land was a significant variable up until the 2008 parsimonious model and this is possibly because irrigated land provides a habitat for mosquito development and water bird foraging in Nepal. Further studies in Nepal should look more closely at the role played by irrigation as it may permit more targeted distribution of JEV vaccine or allow alternative JEV control mechanisms to be considered, such as reducing human proximity to irrigated land. Surprisingly, pig-to-human ratio did not significantly impact JEV transmission despite other studies showing this to be a crucial component to JEV transmission to humans [43], [44]. Furthermore, JE incidence seemed to have a unimodal relationship with pig-to-human ratio. Further study in Nepal would be useful to explain the relationship.

Our model generally did well in predicting JE incidence in Nepal though it underestimated the magnitude of JE incidence in 4 districts. It is still not clear why transmission is particularly high in these areas given we used several explanatory variables in our model. Additional factors, other than those used in this study, may be involved in driving the high level of JE in these districts. When applying our model based on 2005 to the other years (2004, 2006, 2007 and 2008) we see variation in the model fit (i.e. R^2^, adjusted R^2^ and AIC) and the coefficients. This is to be expected as the association between JE transmission and environmental drivers are likely to have statistical noise due year to year variability in JE incidence. In addition, the impact of the immunization campaign may confound the associations.

Our study had 3 major limitations related to spatial demography, ecological fallacy and spatial-temporal analysis of JE transmission.

- Our study utilized the best available data on reported JE incidence at a national scale. However, it is important to recognize that the dataset is unlikely to have captured all JE occurrences in Nepal [20]. There is likely to be some under representation due to limited access to health care, death before seeking health care, health care–seeking behavior in general or reporting to health facilities not included in the Nepal AES surveillance network. Furthermore we did not factor into our analysis the effects of human movement on JE incidence in each district. However, we have no evidence of any systematic biases in these aspects of JE reporting that are likely to have affected the results of our spatial analysis.
- In this study, we did not take scale into consideration (i.e. district size) which may lead to a modifiable areal unit problem (MAUP) which is a sub-class of ecological fallacy [45], [46]. It is possible that if we used a finer areal unit scale in Nepal such as the village development committee (VDCs), we may have obtained different results. For example, the association between JE cases and pig-to-human ratio may have been found to be significant or the model may have better predicted the occurrence of JE in Nepal, particularly in Bardiya, Banke, Dang and Kailali. Still, our results seem to agree with the findings of other studies [13], [38], [40], [41] suggesting similar relationships exist in Nepal. Nonetheless, future analysis will involve using more robust techniques such as conditional autoregressive models to tease out these associations.
- In our regression analysis, we only focused on 2005 data. This was done since this year was an outbreak period with a high number of JE cases (669 cases) and mass vaccination had not been implemented yet, hence not serving as a confounder. While spatial-temporal analysis would have been more informative, we used this study as an initial assessment into the association between JE incidence and climate and land-use/land-cover. Future study will involve spatial-temporal analysis of JE transmission in relation to climate, environment, vaccine coverage and demography.

In conclusion, April precipitation and percentage irrigated land provided the best explanation for JEV transmission spatial pattern in Nepal in 2005. However, with the introduction of the sequential phased mass immunization campaigns that prioritized highest risk districts, it becomes difficult to tease out the associations separate from the impact of the vaccination campaigns. Nonetheless, percentage of irrigated land remains a statistically significant variable influencing JEV transmission despite vaccination efforts. Therefore, increasing our understanding of the interactions between irrigated land and JEV transmission would be valuable in refining our knowledgebase of underlying factors that drive transmission process, such as bird foraging and predator-prey interactions.

Spatial analysis could be an important tool for decision makers to re-prioritize areas which require attention as disease clusters change due to immunization efforts or other changes in agricultural practices and climate change. Our ecological study provides initial but fundamental information that may be useful to Nepal decision makers at the district level in how they address JEV transmission through vaccine distribution or other alternative methods. Further study is needed to refine the findings of this study.

## Supporting Information

Figure S1 **Map of Nepal district by development zone and region.**
(TIF)

Figure S2 **Environmental variables used in full and parsimonious models.** A) Mean May temperature, B) Mean April precipitation, C) percentage grassland cover, D) percent area of irrigated land and E) pig-to-human ratio.
(TIF)

Figure S3 **Predicted smoothed JE incidence (A) and prediction error (B) for the 2005 dataset using the parsimonious model.** The prediction error was calculated as the observed 2005 JE incidence (see Figure 2 in main text) minus the 2005 predicted smoothed JE incidence. The negative values in the prediction error represent overestimation of the model while the positive values represent underestimation of the model. The values were back-transformed from the model using the equation (−1/(y_i_−1))−1 to retrieve the predicted JE incidence.
(TIF)

Table S1 **Pearson's correlation coefficient matrix of variables used in the full model for 2005 JE incidence.**
(DOCX)
