# Supplementary figures and images for: The Spatial Heterogeneity between Japanese Encephalitis Incidence Distribution and Environmental Variables in Nepal
Source: PLoS One. 2011 Jul 21;6(7):e22192. doi: 10.1371/journal.pone.0022192 (PMC3141013; doi:10.1371/journal.pone.0022192)

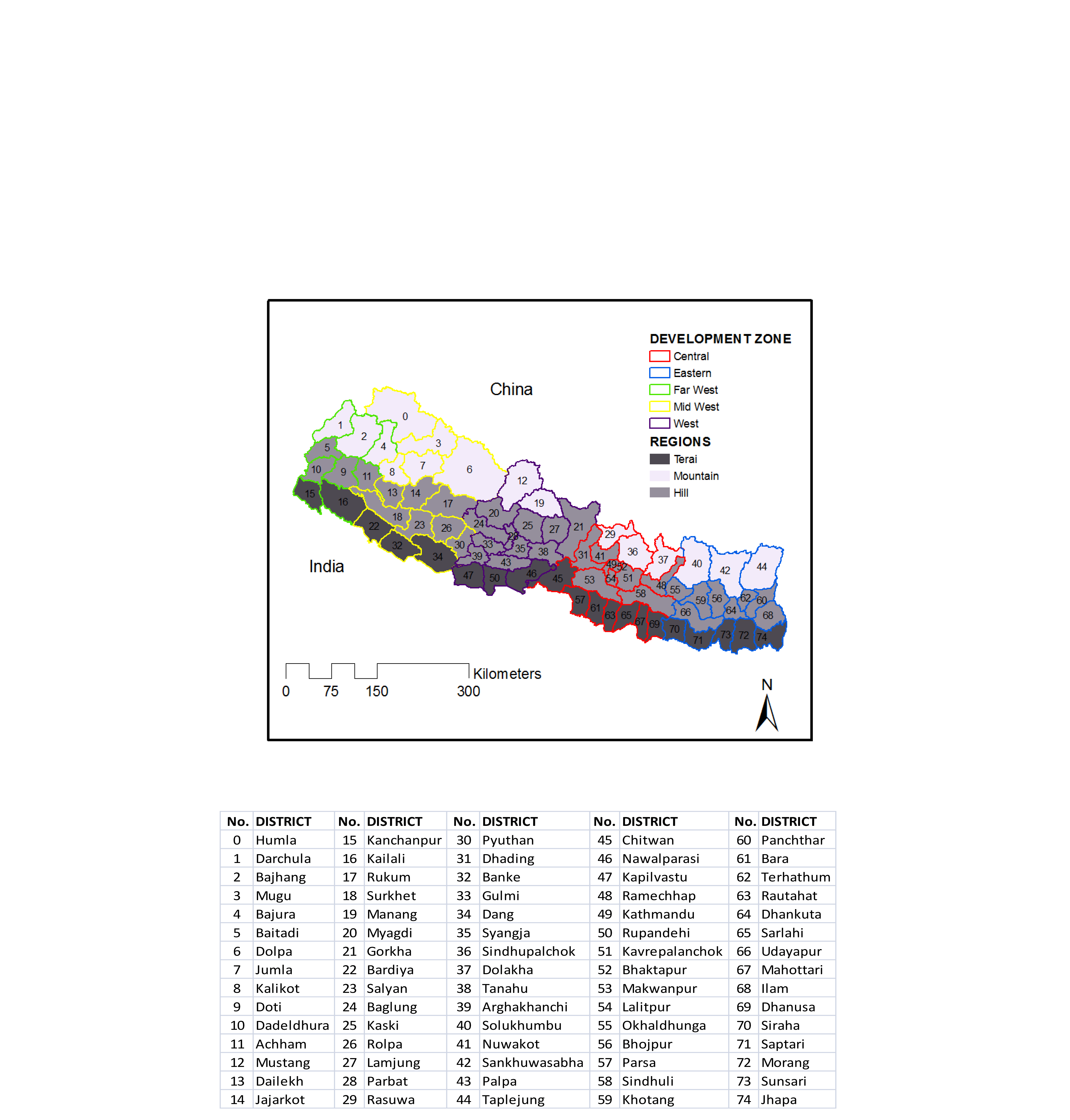

Supplement: Figure S1 — Map of Nepal district by development zone and region. (TIF) [file pone.0022192.s001.tif]

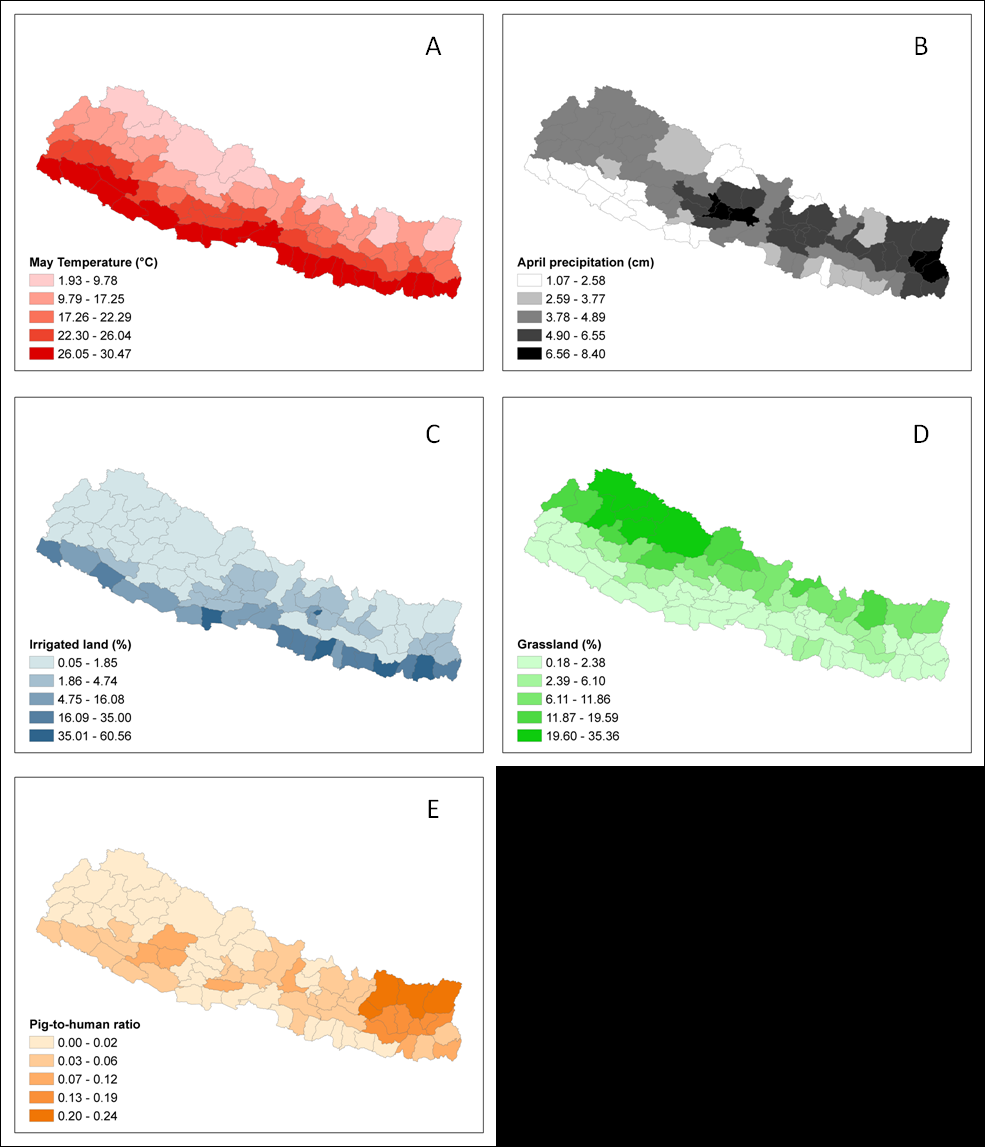

Supplement: Figure S2 — Environmental variables used in full and parsimonious models. A) Mean May temperature, B) Mean April precipitation, C) percentage grassland cover, D) percent area of irrigated land and E) pig-to-human ratio. (TIF) [file pone.0022192.s002.tif]

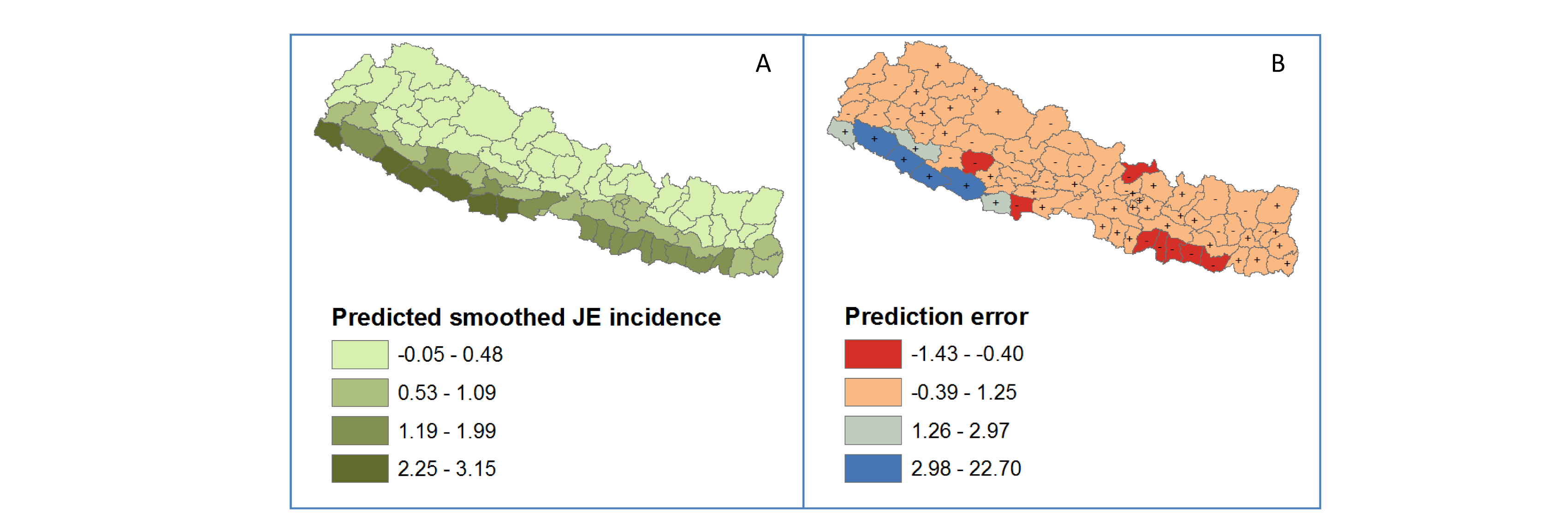

Supplement: Figure S3 — Predicted smoothed JE incidence (A) and prediction error (B) for the 2005 dataset using the parsimonious model. The prediction error was calculated as the observed 2005 JE incidence (see Figure 2 in main text) minus the 2005 predicted smoothed JE incidence. The negative values in the prediction error represent overestimation of the model while the positive values represent underestimation of the model. The values were back-transformed from the model using the equation (−1/(yi−1))−1 to retrieve the predicted JE incidence. (TIF) [file pone.0022192.s003.tif]
