# Supplementary material for: The Spatial Heterogeneity between Japanese Encephalitis Incidence Distribution and Environmental Variables in Nepal
Source: PLoS One. 2011 Jul 21;6(7):e22192. doi: 10.1371/journal.pone.0022192 (PMC3141013; doi:10.1371/journal.pone.0022192)
Supplement: Table S1 — Pearson's correlation coefficient matrix of variables used in the full model for 2005 JE incidence. (DOCX) [file pone.0022192.s004.docx]

| **Table S1.** Pearson correlation coefficient matrix of variables used in the full model for 2005 JE incidence | | | | | | | | |
| --- | --- | --- | --- | --- | --- | --- | --- | --- |
|  | ***JE*** | **May*-t*** | **Apr*-p*** | ***Irrigated*** | ***Pig-human*** | ***Deciduous*** | ***Grasslands*** | ***Croplands*** |
| ***JE*** | 1 | - | - | - | - | - | - | - |
| **May*-t*** | .730^**^ | 1 | - | - | - | - | - | - |
| **Apr*-p*** | -.434^**^ | -.151 | 1 | - | - | - | - | - |
| ***Irrigated*** | .681^**^ | .802^**^ | -.100 | 1 | - | - | - | - |
| ***Pig-human*** | -.212 | -.083 | .349^**^ | -.079 | 1 | - | - | - |
| ***Deciduous*** | .442^**^ | .568^**^ | -.127 | .582^**^ | .236^*^ | 1 | - | - |
| ***Grasslands*** | -.640^**^ | -.842^**^ | -.055 | -.721^**^ | .021 | -.407^**^ | 1 | - |
| ***Croplands*** | .609^**^ | .681^**^ | -.414^**^ | .736^**^ | -.332^**^ | .449^**^ | -.442^**^ | 1 |
| **Correlation is significant at the 0.01 level (2-tailed), *Correlation is significant at the 0.05 level (2-tailed)  *JE* =JE incidence,  *t* = mean temperature, *p* = mean precipitation, *Irrigated* = natural log of percentage of irrigated land per district, *Pig-human* = pig-to-human ratio, *Deciduous =* natural log of percentage deciduous tree cover per district, *Grassland* = natural log of percentage grassland cover per district, *Cropland* = natural log of percentage cropland cover per district  ^†^2005 district-level JE incidence was smoothed using Spatial empirical Bayes smoothing and transformed by taking negative reciprocal | | | | | | | | |
